# Depression, Estrogens, and Neuroinflammation: A Preclinical Review of Ketamine Treatment for Mood Disorders in Women

**DOI:** 10.3389/fpsyt.2021.797577

**Published:** 2022-01-18

**Authors:** Collin Gagne, Alexandre Piot, Wayne G. Brake

**Affiliations:** Department of Psychology, Centre for Studies in Behavioural Neurobiology Concordia University, Montreal, QC, Canada

**Keywords:** microglia, glutamate, neuroinflamation, sex differences, estradiol (17ß-estradiol)

## Abstract

Ketamine has been shown to acutely and rapidly ameliorate depression symptoms and suicidality. Given that women suffer from major depression at twice the rate of men, it is important to understand how ketamine works in the female brain. This review explores three themes. First, it examines our current understanding of the etiology of depression in women. Second, it examines preclinical research on ketamine's antidepressant effects at a neurobiological level as well as how ovarian hormones present a unique challenge in interpreting these findings. Lastly, the neuroinflammatory hypothesis of depression is highlighted to help better understand how ovarian hormones might interact with ketamine in the female brain.

## Ovarian Hormones & Depression

Sex differences in mood disorder prevalence are common and can emerge during adolescence (1, 2). Depression rates are nearly two-fold higher in women than men aged 14–25 (3). Yet, these sex differences decrease with age, such that depression prevalence among men and women tend to be similar around late adulthood (4). A cross-national comparison revealed that women aren't at even more increased risk for depression in countries where sex inequalities are more marked (5) although some caveats were noted in the analysis. This could indicate that potentially confounding factors are not responsible for the differences in prevalence rates. Other findings suggest that sex differences in depression prevalence may be due to inherent factors such as sex steroid hormones in combination external factors such as increased exposure to early life adversity and gender inequities (6).

Estrogens are a family of ovarian hormones, often collectively referred to as estrogens (E), of which there are three subtypes, viz. estrone, estradiol, and estriol. The most potent estrogen, in terms of its affinity for estrogen receptors in both rodents and humans, is 17β- estradiol (7). The ovaries synthesize and release the bulk of E in a cyclical manner, peaking during ovulation. Yet, in both sexes, the adrenal glands as well as neurons and glial cells in the central nervous system also synthesize and release E, albeit in much smaller amounts.

Like E, another ovarian hormone, progesterone (P) is mostly synthesized in the ovaries in a cyclical manner, specifically by the corpus luteum, a temporary gland formed during the ovulatory phase of the human menstrual cycle and after the proestrus phase of the rat estrus cycle. The estrus cycle is the term for the female sex steroid cycle in most non-primate mammals. P is also produced by the placenta once it's formed during gestation, and the adrenal glands, and again by cells in the central nervous system where it's formed by steroidogenesis from cholesterol in both males and females (8, 9).

Douma et al. (10) suggest that a reduction of E levels, a continued deficit of E, or changes in E levels throughout the menstrual cycle are correlated with mood-related distress (11). This idea is also supported by multiple studies demonstrating a link between post-partum depression (PPD) and E levels (12–14). PPD is the emergence of a depressive episode after parturition (i.e., giving birth). This disorder affects between 7 and 20% of women who have given birth and is thought to be caused by fluctuations in E and P levels [see Schiller et al. (15) for review]. While some studies have been unsuccessful in establishing a relationship between hormone levels and PPD in women (16, 17) others have found that treating PPD with estradiol alleviates symptoms (12, 18). Animal models have shown that E and P removal can lead to depressive-like behaviors (19, 20) and hormone replacement can ameliorate these behaviors. It would be wrong, however, to perceive depression as simply linked to a decrease in ovarian hormones in females. Indeed, premenstrual dysphoric disorder (PMDD) illustrates how an increase, rather than decrease, in ovarian hormones is associated with depressive symptoms (21, 22).

PMDD affects 3–8% of premenopausal women and can repeat monthly. Symptoms, such as depressive mood, occur in the luteal phase when P and E are on the rise (23). Importantly, the hormonal profiles of women with PMDD are indistinguishable from those without (24). This suggests that PMDD sufferers are perhaps more reactive to ovarian hormones at a neurobiological level. Furthermore, early menarche, i.e., first menstruation, in girls (prior to 11.5 years of age) has been associated with an increase in E and an increase in depressive disorders in adolescence (25). Other evidence demonstrating an ovarian hormone-depression link comes from menopause and hormone replacement therapy (HRT) in older women.

In the past a substantial proportion of post-menopausal women making use of HRT at one point in their lives, with some estimates being as high as 38% (26). Today, due to efforts demonstrating associations between HRT and various negative health outcomes, post-menopausal women will typically receive HRT between the ages of 50–59 (or <10 y post-menopause) and for shorter time periods (27–29). In such cases HRT typically consists of E, but progestins, such as P, can also be administered either alone or in tandem with E (26, 30). The effects of HRT on depressed mood during menopause are promising. One meta-analysis of 26 studies reveals that E have a considerable effect size (*d* = 0.69) toward decreasing depressed mood, whereas P solely or in tandem with E was associated with less potent effects (31).

Considering the evidence, it is clear that ovarian hormones may worsen or improve depressive symptoms, but the direction of the relation can change due to various factors. The fluctuating nature of the menstrual cycle, as well as the physiological and cellular changes it entails, renders simplistic generalizations unworkable. To better understand the role of the menstrual cycle in depression one should consider how hormone fluctuations affect the expression of depressive symptoms in tandem with cellular processes, particularly in the brain. To do so, we must firstly consider the neurobiological basis of depression.

## Hypotheses of Depression

There are multiple models suggesting what may cause depression, none of which are mutually exclusive, highlighting the complexity of the underlying etiology. One of the more prolific, but dated, theories of depression is the monoamine hypothesis. Put simply, this theory suggest that major depressive disorder (MDD) is caused by depleted levels of the monoamine neurotransmitters dopamine, norepinephrine, or serotonin; or some combination of these (32, 33). While there is evidence in support this theory, for example monoamine depletion diets (34), there is plenty that contradicts it, i.e., the slow onset and moderate efficacy of antidepressants targeting monoamines (35–39).

Another hypothesis, the glutamate hypothesis of depression, posits that MDD is linked to altered glutamate transmission and metabolism in the brain. Studies indicate that there is an increase of glutamate in the plasma of depressed patients compared to healthy controls (40, 41). This can be reversed with the administration of SSRIs (40, 42). This idea suggests that excessive glutamate transmission, associated with a decrease in synaptic clearance by neighboring glial cells, results in cellular toxicity and a reduction in brain volume (43). Brain imaging studies show that individuals suffering from MDD and anxiety-related disorders have smaller hippocampal and prefrontal cortical volumes (44–47). This theory attributes such atrophy to inflammatory glial cells transitioning to an inflammatory state, causing lowered synaptic clearance, neurotoxicity, and phagocytosis of healthy neurons. Moreover, stress, a major predictor of depression (48, 49), has been shown to increase synaptic glutamate in the hippocampus [HPC; (50)] and prefrontal cortex of rats (51). It is proposed that while glial cells carry out the inflammatory response, glutamate metabolism and synaptic clearance may decline, resulting in modified glutamate transmission which could contribute to the emergence of depression. *In vivo* studies have found lower levels of glutamate metabolites in the cingulate (52) and frontal regions (53) of the prefrontal cortex. These alterations in glutamate metabolism have been intricately associated with depression and treatment resistance (45).

## The Neurotrophic Theory of Depression

Another idea is that neurodegeneration and atrophy is of paramount importance to depression's etiology, also known as the neurotrophic hypothesis of depression. Stress can, under some circumstances, cause neuronal apoptosis, dendritic atrophy, and decreases in trophic factors in the HPC, where atrophy and a lower volume is often observed among depressed individuals (54–56). Santarelli et al. (57) showed that induction of hippocampal neurogenesis is necessary for antidepressants to produce behavioral effects in a mouse model of depression. Furthermore, electroconvulsive therapy (ECT), causes increases in trophic factors in the HPC of rats (58).

Brain-derived neurotrophic factor (BDNF) is the most well-studied trophic factor in the context of antidepressants and research supports the idea that BDNF plays an important role in MDD. Post-mortem research shows that the HPC of humans with MDD had lowered levels of BDNF, while those who were taking antidepressants at the time of their death had higher levels (59). Similar effects have been observed in both the HPC and prefrontal cortex (PFC) of suicide victims in comparison to controls (60, 61). It has been proposed that BDNF levels, in both serum (62–65) and plasma (66, 67) are a viable biomarker for MDD and even prescription adherence. This is supported by studies showing that BDNF levels are increased when humans (63, 68–71), or rodents (58, 72) are administered antidepressants. When BDNF itself is administered, either directly to the HPC (73, 74), or peripherally (75), antidepressant-like effects are observed in rodents. Work by Shirayama et al. (73) showed that these effects last for as long as 10 days after the infusion, long after the protein has degraded, suggesting that BDNF triggers mechanisms which sustain its effects on plasticity. Finally, other, non-pharmacological antidepressant interventions, such as exercise (76) and ECT (58) have been shown to increase central BDNF levels in rodents.

## The Neuroinflammatory (Cytokine) Theory of Depression

The neuroinflammatory model of depression, also known as the cytokine hypothesis, is comprehensive in the sense that it ties together both the monoamine and neurotrophic theories of depression. To put it simply, this theory posits that depression is caused by inflammatory processes (77–80) that involve microglia.

Microglia, a type of macrophage, can act as part of the brain's immune response and make up approximately 10% of cells in the central nervous system. Microglia are responsible for many processes such as inducing apoptosis in nearby neurons and synaptic pruning, both of which are necessary for healthy brain development and maintaining homeostasis (81–84). A microglial response to threats could entail the release of reactive nitrogen and oxygen species to cause oxidative stress and/or apoptosis in infected neurons (79). Microglia also release small proteins known as cytokines, which can be pro-inflammatory such as; interleukin (IL)−1, 2, 6, and 18 (83, 85, 86), tumor necrosis factor (TNF) α (87) and interferon (IFN) γ (88), or anti-inflammatories, such as IL-4, 10, and 13 (89–91). One signal that microglia use to extend their processes toward a target neuron is adenosine triphosphate [ATP; (92)]. It was found that N-methyl-D-aspartate receptor (NMDA) receptor activation mediates this ATP release. However, it is hypothesized that this mechanism of microglial surveillance has evolved because ATP is also released during apoptosis, thus acting as a “find me” signal for all immune cells (92–94). Importantly, microglia often engage in phagocytosis, when they extend a cup-shaped process to engulf a given target (95). The idea that microglia-mediated neuroinflammation contributes to the etiology of depression is not a new one (96, 97). Sickness behavior, an adaptive behavioral strategy where motivational state is reorganized to optimize coping with illness (98) may be related to depressive behaviors. While sickness behavior clearly demonstrates how depressive behavior is elicited by immune challenges, the neuroinflammatory hypothesis of depression posits that stress is the first step leading to depression (79).

Common psychosocial stressors, such as lack of social support or exam stress have been shown to increase the production and release of pro-inflammatory cytokines (99, 100). Researchers have shown that the Trier social stress test elicits increases in circulating IL-6 (101) and Il-1β (102). Aschbacher et al. (102) showed that peripheral immunoreactivity to stress even predicts future depressive symptoms in a cohort of post-menopausal women and menopause has been associated with an increase in pro-inflammatory cytokines (103). Studies of older adults have also found that levels of peripheral pro-inflammatory IL-1 family cytokines were positively correlated with future depressive symptoms (104, 105).

van den Biggerlaar et al. (105) demonstrated that *ex vivo* whole blood cytokine production in response to LPS (i.e., lipopolysaccharide, which is used to generate an immune response) administration also predicted future depressive symptoms. Stress-induced increases in pro-inflammatory cytokines, which are increased in people with depression (77–80, 97, 106–108), have been positively correlated with severity of depressive symptoms (109). Pro-inflammatory cytokines affect the CNS through several mechanisms which are hypothesized to then cause depression.

Centrally administered LPS, which causes microglia to release pro-inflammatory cytokines, has been shown to reduce rat hippocampal neurogenesis (110). In fact, the number of activated microglia, indicated by CD68 labeling, was inversely correlated with new neurons. These findings showed that new hippocampal neurons simply do not survive around activated microglia. Conversely, inhibiting microglial activation, via minocycline, gives rise to increases in new neurons. The damaging effects of activated microglia to their neighboring neurons are likely mediated by cytokines such as IL-1β and IL-6 (111, 112). Monje et al. (113) also confirmed that microglial activation is negatively associated with neurogenesis. This work ties together both the neuroinflammatory and neurodegenerative hypotheses of depression.

Common to both the monoamine and neuroinflammatory hypotheses of depression is the kynurenine pathway, which consists of the following reactions: Tryptophan, an essential amino acid and precursor for the monoamine neurotransmitter serotonin [5-HT; (114, 115)], is catabolized into kynurenine by the enzyme, and rate-limiting factor (116), indoleamine 2,3-dioxygenase [IDO; (117)]. Kynurenine is then converted into either quinolinic acid (QUIN), a neurotoxic NMDA-receptor agonist, or kynurenic acid, an NMDA, α-amino-3-hydroxy-5- methyl-4-isoxazolepropionic acid (AMPA), and kainate receptor antagonist, which has been also shown to be protective against excitotoxicity (118, 119). The kynurenine pathway is controlled by the immune system, with the synthesis of certain downstream metabolites, like QUIN, occurring within microglia (120, 121). When this pathway is more actively engaged, for example due to pro-inflammatory cytokines which induce IDO (122–124), less tryptophan is available for 5-HT synthesis (125–129). This directly relates the neuroinflammatory and monoamine hypotheses of depression. Indeed, this process resulting in 5-HT depletion has been implicated in the etiology of depression for decades (130).

## Ketamine: A Novel Antidepressant

The first administration of ketamine to humans occurred at Jackson prison in 1964 soon after its synthesis (131). Decades later, it was discovered to have rapid antidepressant effects among individuals with MDD (132). Historically, ketamine was largely used as an animal anesthetic and sedative and still is today. Additionally, sub-anesthetic doses of ketamine have robust analgesic effects while having little impact on the respiratory system in comparison to opioids, like morphine or fentanyl (133). Ketamine's neuroprotective and anti-inflammatory effects led to its use in emergency medicine for burn patients and its calming and dissociative effects have led it to be administered to patients who are suicidal or in shock (134, 135). When Berman et al. (132) first reported ketamine's antidepressant effects among individuals with MDD, it was largely ignored. It was another 5 years before the study was replicated (136), prompting the original study to become highly cited. Since Zarate et al. (136) replicated Berman's work, ketamine's antidepressant effects have been well-established. A clinical trial was initiated at the National Institute of Mental Health (137), and the medication Spravato (S- ketamine) by Johnson & Johnson was approved by the U.S. Food and Drug Administration as a breakthrough treatment in March 2019, although only indicated for TRD. The neurobiological mechanisms responsible for ketamine's antidepressant effects are not yet fully understood, but several theories have been postulated.

## The Glutamate Burst Hypothesis of Ketamine Action

The glutamate burst hypothesis [a.k.a. the disinhibition hypothesis; (138)] posits that ketamine effectively reverses the synaptic and dendritic spine atrophy, notably in the PFC and HPC, known to be associated with stress and depression. Central to this hypothesis is the NMDA receptor (NMDAR).

The NMDA receptor is composed of GluN1 and GluN2 subunits, typically two of each, forming a heterotetramer. The complex can also contain GluN3 subunits (139, 140). The subunits are further divided into isoforms (GluN1A, GluN1B, GluN2A, etc.). NMDARs can be thought of as both ligand and voltage gated. For the channel to open, glycine (or D-serine) must bind to GluN1, glutamate must be bound to GluN2 subunits, and the neuronal membrane must be depolarized to a sufficient degree such that the Mg^2+^ blockade at the center of the receptor pore is removed. Only when these conditions are met does the channel become permeable to Na^+^, K^+^, and importantly Ca^2+^ ions, producing a plethora of intracellular second messenger cascades (141). Ketamine is a non-competitive NMDAR antagonist, but like other NMDAR ligands, its pharmacological interactions vary based on subunit composition. Ketamine, MK-801, and phencyclidine, block the NMDAR channel at the same site, and are more likely to do so when the receptor is composed of GluN1/GluN2A or GluN1/GluN2B subunit configurations (139).

Gerhard et al. (142) suggest that ketamine leads to a burst of glutamatergic transmission which ultimately reverses synaptic and dendritic spine atrophy, and that this occurs in five steps. The glutamate burst hypothesis: a proposed mechanism of action for ketamine. First, ketamine blocks NMDARs located on inhibitory GABA interneurons. The same research group showed this occurs preferentially through blocking NMDARs on GABA interneurons in mice, specifically in the mPFC (143). This disinhibition occurs preferentially on GABA interneurons likely due to their higher frequency of firing compared to pyramidal neurons. Faster firing allows for NMDARs to be freer of their Mg^2+^ ion blockade, granting ketamine greater access to the NMDA channel to inhibit its opening (138). This idea is supported by both human research, by evidence showing ketamine increases overall PFC activity in healthy individuals (144), and rodent research, by evidence that MK-801, specifically increases pyramidal neuron-firing in the PFC (145). Second, by blocking the NMDA channels on GABA interneurons, ketamine reduces tonic firing of these interneurons, subsequently resulting in a disinhibition of glutamate neurons, and a burst in glutamate release. Gerhard et al. (143) showed that ketamine's blockade of NMDARs on GABA interneurons in the mPFC caused a disinhibitory net effect in the form of an increase in excitatory postsynaptic currents in layer V primary neurons. Furthermore, the effect appeared to occur through GluN2B-containing NMDARs in male mice only, as was demonstrated via knockdown and genetic deletion models in mice, suggesting sex-differences at a molecular level (143). Third, as a result of a presynaptic glutamate burst, AMPA receptors are activated and upregulated on postsynaptic sites of PFC pyramidal neurons, resulting in greater depolarization of the postsynaptic membrane. Nearby post-synaptic voltage-gated calcium channels open and an influx of calcium occurs. Fourth, BDNF is released by the postsynaptic neuron and binds to tropomyosin receptor kinase B (TrkB) receptors, also on the postsynaptic neuron, initiating several intracellular second messenger cascades. TrkB receptors auto-phosphorylate, allowing them to affect cellular function for extended periods of time. This could explain how centrally administered BDNF produces prolonged antidepressant effects (73). Lastly, targets of rapamycin complex 1 (mTORC1) signaling proteins are rapidly phosphorylated, increasing spine density in the PFC via proteins like postsynaptic density protein (PSD) 95, and synapsin. The importance of the mTORC1 signaling cascade is illustrated by paradigms which block mTORC1 via rapamycin, reducing ketamine's antidepressant-like effects in animal models (138, 142, 146, 147).

Furthermore, convergent pathways which increase synaptogenesis and protein synthesis (such as the mTOR pathway) have been proposed as potential mechanism for the antidepressant properties of psychedelic drugs as such lysergic acid diethylamide, psilocybin, or dimethyltryptamine in addition to ketamine [reviewed in Aleksandrova and Phillips (148)].

## Beyond the Glutamate Burst Hypothesis

Another hypothesis of ketamine's mechanism of action–proposed, in part, by several research groups–holds that a glutamate burst is not necessary (149–151). Instead, ketamine's NMDA antagonism blocks spontaneous miniature excitatory postsynaptic currents at rest, which are mediated by NMDA activation. It is suggested that it is the deactivation of eukaryotic elongation factor 2 (eEF2) kinase (a.k.a. CaMKIII), not the activation of mTORC1, at the root of this effect. Deactivation of eEF2 kinase, via ketamine administration, was shown to induce BDNF and dendritic protein translation, including AMPA subunits, in mouse models. This paradigm suggests that NMDA receptor activity at rest allows for eEF2 kinase to chronically phosphorylate eEF2, which then suppresses translation. It is suggested that acutely blocking NMDA, via ketamine, stops eEF2 phosphorylation via eEF2 kinase deactivation, which produces antidepressant-like effects (149–151).

A third ketamine hypothesis (152) posits that NMDA antagonism, and subsequent second-messenger cascades, is not central to ketamine's antidepressant effects. This is because ketamine's R enantiomer produces greater antidepressant-like effects in rodent models than the S enantiomer, despite R-ketamine having an almost four-fold lower affinity (Ki = 2.57) for the NMDA receptor than S-ketamine [Ki = 0.69; (153)]. Research supporting this hypothesis shows that ketamine's antidepressant effects actually occur via its metabolites, specifically 2R and 6R hydroxynorketamine (2R−6R HNK), acting on AMPA receptors. Administering deuterated ketamine, which is not metabolized into other compounds due to a change in its molecular structure, produces no antidepressant-like effects in rodent models. Furthermore, administering 2R-6R HNK with 2,3-dihydroxy-6-nitro-7-sulfamoyl- benzo(f)quinoxaline-2,3-dione (NBQX), an AMPA receptor antagonist, causes no antidepressant-like effects, whereas, without NBQX, the 2R-6R HNK metabolites produces the most potent effects compared to other metabolites (152). Proponents of this theory, therefore suggest ketamine's mechanism of action lies with its ability to activate AMPA receptors (152, 154, 155).

## Ketamine in Females

For the above hypotheses of ketamine's antidepressant effects, most of the data has been derived from studies on males. As one might expect, there is a growing body of evidence which suggests that these theories do not hold the same for females. In support of the glutamate burst hypothesis, male rodents with social isolation stress (SIS) have a decrease in sucrose preference, a measure of anhedonia, as well as decreases in medial PFC (mPFC) neuron spine density, PSD- 95, and synapsin. Three hours after a single ketamine infusion all of these effects are reversed. In females, SIS also leads to a decrease in mPFC spine density, PSD-95 and synapsin, yet no changes in sucrose preference and none of these effects were reversed by ketamine (156). Furthermore, ketamine induces hippocampal glutamate in males but not females, and induces aspartate in the mPFC in females but not males (157). All this evidence is contrary to the glutamate burst hypothesis and suggests that the glutamatergic system in females reacts to ketamine differently than in males. Interestingly, female rats require half the minimum dose that males do to produce antidepressant effects, but this effect is only seen when both E2 and P are present (158, 159). This suggests that there are sex differences in the mechanism of action of ketamine and that ovarian hormones may play a role in how it acts in the female brain.

Carrier and Kabbaj (158) have shown that ketamine does not decrease eEF2 in the HPC and PFC of female rats, whereas this decrease has been shown to occur consistently in males (149–151, 158). One theory, supported by evidence in both males and females, places primary importance on ketamine's metabolites. However, while both 2R and 6R-HNK are more abundant in females than males, so too are 2S and 6S-HNK (152, 160). This evidence may explain the sex differences in terms of sensitivity to ketamine's antidepressant-like effects in rodents, but it only muddies the waters in terms of how ketamine may be exerting sex-specific effects. Making the story even less clear, the few meta-analyses conducted among clinically treated individuals have revealed no sex differences in ketamine's antidepressant effects (161), or a slightly higher sensitivity among males, but only at 7 days post-infusion (162). Although more research emphasizing potential sex differences must be carried out to replicate these findings, this suggests that there exists no difference between the sexes among humans in terms of response to ketamine.

When looking to the more clinically relevant S-ketamine, researchers have shown it is eliminated at a faster rate in women than in men, although this investigation was performed in the context of analgesia (163). In fact, there is a dearth of research investigating clinical sex differences when it comes to the efficacy of S-ketamine in comparison to racemic ketamine. One non-inferiority clinical trial (*N* = 63) looked to compare efficacy between the two molecules and found there were no differences between S-ketamine and racemic ketamine in improving depression. Sex differences were not analyzed but given the size, and results, of the study it is unlikely that sex might have accounted for clinically relevant effects (164). Following this trend, Bahji et al. (165) conducted a meta-analysis to explore whether any differences might exist between the two molecules and examined 24 studies (*N* = 1,877), but failed to include sex-differences in any analyses or discussion. Importantly, the authors did find that IV racemic ketamine alleviates the symptoms of depression more effectively than intranasal S-ketamine. The literature is lacking a critically important meta-analysis of sex-differences among ketamine clinical trials. Nevertheless, a much more recent and comprehensive theory on the etiology of depression may be the missing link in explaining ketamine's effects in the female brain.

## Microglia, Neuroinflammation, and Ketamine

Recently, the kynurenine pathway was suggested as a mechanism through which ketamine might affect neurodegeneration via microglia-mediated neuroinflammation (166). Several studies now have shown that LPS induces QUIN in microglia (167–169). Moreover, higher levels of neurotoxic QUIN was found in the cerebrospinal fluid of suicide victims (170). Furthermore, QUIN was increased specifically in microglia in the post-mortem brains of severely depressed individuals (171).

Establishing ketamine's relevance to this paradigm-shift in our understanding of the neurobiology of depression, Walker's et al. (172) showed that ketamine reverses LPS- induced depressive-like behavior in rodents. What's more, this effect occurred when ketamine was given 10 h after LPS administration, giving the inflammatory and kynurenine pathways enough time to be activated. Pre-treatment with ketamine blocked LPS-induced depressive-like behavior from developing. Interestingly, ketamine had no impact on LPS-induced inflammatory activation in the form of plasma cytokine levels. The researchers hypothesized that ketamine, an NMDA antagonist, was instead blocking QUIN's ability to act as an NMDA-receptor agonist. As mentioned previously, a requirement for ketamine's antidepressant effects to take hold is the upregulation of AMPA-mediated glutamatergic neurotransmission, likely due to ketamine's metabolites (152, 154, 155). Their hypothesis was confirmed via administering NBQX 15 min before ketamine, restoring depressive-like behavior (166, 172).

Verdonk et al. (173) were the first to show that microglia are a direct target of ketamine and, specifically, the production of QUIN within microglia. In a mouse model of LPS-induced depression ketamine induced changes in microglia resulting in a neuroprotective phenotype. They observed that ketamine reversed the LPS-induced increase in QUIN in brain parenchyma, whereas ketamine had no effect on QUIN in control animals. Translating this work to a clinical perspective, the researchers also tested kynurenine pathway metabolite concentration in 15 individuals with TRD in response to ketamine. They found that the KYNA:QUIN ratio before the first ketamine infusion to be a significant predictor of final Montgomery–Åsberg Depression Rating Scale (MADRS) score. This effect was driven primarily by QUIN plasma levels. QUIN concentrations before each ketamine infusion were the best predictor of ketamine efficacy and were the only significant predictor of relative change (i.e., before and after ketamine) in MADRS scores. These findings by Verdonk et al. (173) confirm the hypothesis of Walker et al. (172) that ketamine directly affects microglial QUIN levels, in both mice and humans, resulting in protection from neuroinflammatory processes, and that the degree to which ketamine impacts QUIN levels correlates with relief of depressive symptoms in humans.

**Figure 1:**
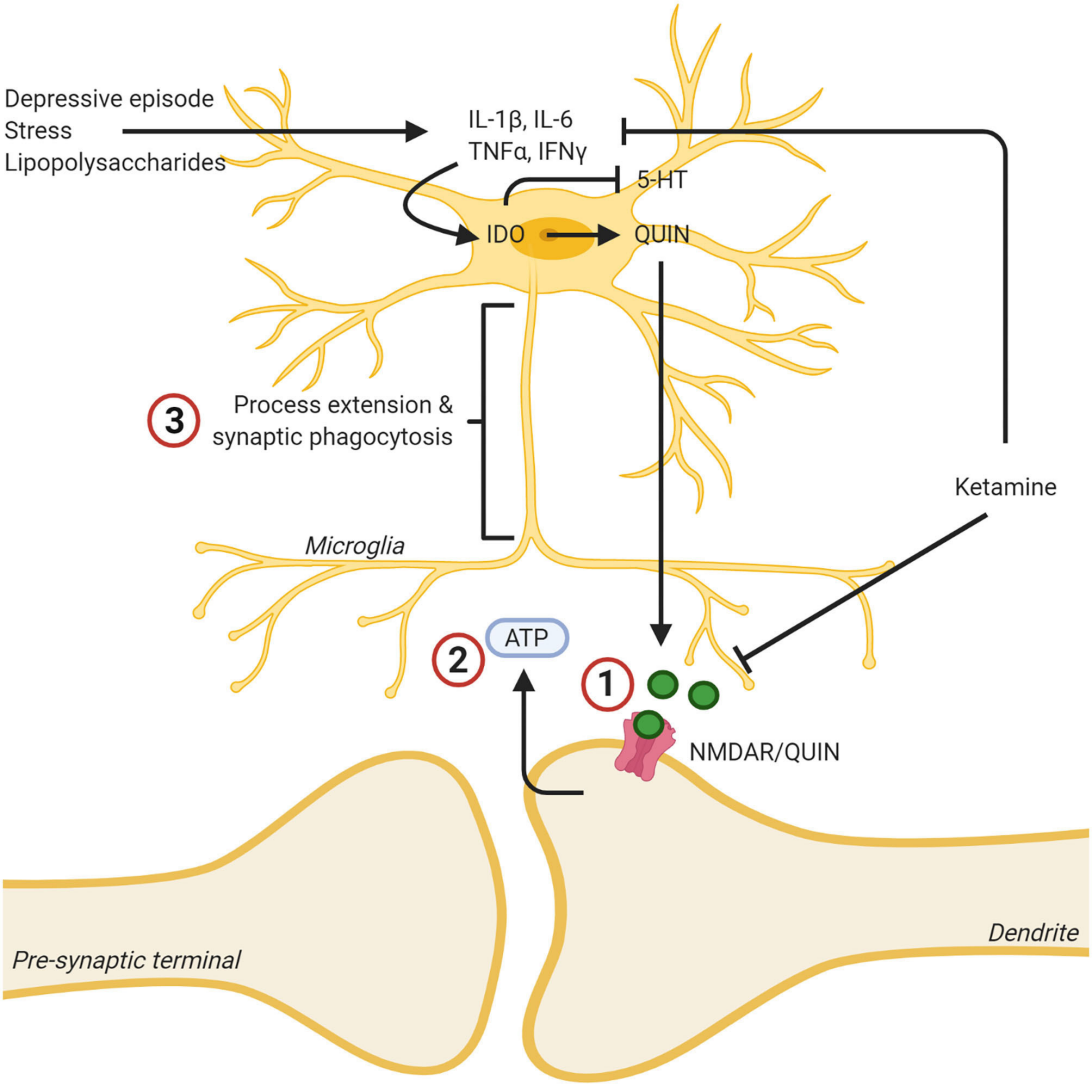
Ketamine's effects on the kynurenine pathway leads to lowered levels of neurotoxic quinolinic acid (QUIN) via inhibition of cytokine production, and lowered effects of QUIN/NMDA binding by blocking the NMDA receptor. (1) Ketamine binding blocks the NMDA receptor, reducing the neurotoxic effects of QUIN released from microglia. (2) This decreased neurotoxicity decreases ATP release from the synapse. (3) As a result, the microglia no longer extend toward this “find me” signal and the inflammatory reaction is dampened, and the microglia assumes a less reactive state. In addition, ketamine was found to mitigate the reaction of microglia to inflammatory stimuli such as stress or LPS and decreased the production of proinflammatory cytokines. Created with BioRender.com.

Further linking ketamine to inflammatory processes, researchers have shown that ketamine reverses the effects of LPS, in the form of pro-inflammatory cytokine release, in microglia *in vitro* (174). This makes sense, given that others have shown that NMDA and LPS have very similar effects on microglia. Both produce a release in pro- inflammatory cytokines and the adoption of a more amoeboid-like shape, indicating a more active state (175). NMDA receptors have been implicated in cytokine-induced neurotoxicity in previous research as well. When Chao et al. (176) administered both IL-β and TNF-α to *in vitro* fetal brain cells, they noticed a marked increase in neuronal injury. When these cytokines were administered in conjunction with MK-801, the increase in neuronal injury was ablated. Recently, researchers showed that ketamine reduced pro-inflammatory cytokines, microglia phagocytic markers in the rat HPC, and depressive-like behavior in a rat model (177), see Figure 1.

Much of the evidence presented thus far, points to microglia playing a pivotal role in ketamine's antidepressant effects. Ketamine's NMDA-antagonism make it capable of stopping neuronal ATP release, stopping microglia from potentially damaging nearby neurons, as previously discussed (92). Ketamine reduces the neurotoxic effects of QUIN being produced within microglia (173). Ketamine also reduces the microglial response to otherwise activation-inducing molecules like LPS (174) and stops microglial production of pro-inflammatory cytokines (177). Unfortunately, the vast majority of this research has been conducted using the male sex. Similarly, the theoretical bases which guide such experiments has also, historically, used the male sex. An important consequence of this, beyond the inability of generalizing these findings to 50 percent of the population, is the lack of understanding how ketamine might interact with ovarian hormones. Fortunately, extensive research has been conducted investigating the role of estrogens on microglia.

## Ovarian Hormones and Neuroinflammation

The classical mechanism through which lipid-soluble hormones like E2 act on neurons and glia is via nuclear estrogen receptors (ERs). Hormones like E2 diffuse across the cell membrane passively and bind to nuclear receptors in the cytoplasm. These ligand-bound receptors dimerize with other ligand-bound receptors, forming a hetero or homodimer (178–180), and translocate to the nucleus where they act as transcription factors. Nuclear estrogen receptors were thought to only function this way, but more recently membrane-bound estrogen receptors have been discovered, such as G- protein-coupled estrogen receptor 1 (GPER1), and act more rapidly (181). Estrogens, as a whole, have been found to be neuroprotective toward multiple pathologies, most notably those in which microglia are implicated, such as Alzheimer's disease (182–186) and multiple sclerosis (187–190).

One way that E2 impacts microglia is via the nuclear estrogen receptor (ER) α. Researchers have found that systemic administration of E2 reduces LPS-induced microglia activity in a dose-dependent manner by decreasing the expression of proteins associated with phagocytosis, by inhibiting morphological changes, and by inhibiting cell migration. ER knockout mouse models demonstrated that ERα is responsible for these effects. For example, microglia activity was unaffected by the absence or presence E2 administration in ERα-null mice (191). Previous research by Bruce-Keller et al. (192) showed that, in a dose-dependent manner, E2 attenuates microglia phagocytosis, and the release of superoxide, a neurotoxic free radical. These effects were mediated by the phosphorylation of mitogen-activated protein kinase (MAPK). Additionally, Vegeto et al. (193) also found that E2 reduces the buildup of free-radicals, specifically, nitrous oxide, in microglia.

Another way that E2 can impact microglia is via GPER1. E2 binding to GPER1, located predominantly on the endoplasmic reticulum, but also the Golgi apparatus and nuclear membrane, results in the mobilization of intracellular calcium, and the production of nuclear phosphatidylinositol 3,4,5-trisphosphate [PIP3; (194)]. PIP3 is an effector of multiple downstream signaling proteins, particularly the protein kinase AKT which plays a crucial role in several cellular processes, such as cell survival (195–197) and proliferation (198, 199). Zhao et al. (200) were the first to show that GPER1 mediates E2's anti-inflammatory effects on microglia in a rat model of cerebral ischemia. Both E2, or the GPER1 agonist, G1, alone were able to attenuate LPS-induced increases in pro-inflammatory TNF-α and IL-1β. Co-administration of the GPER1 antagonist, G15, with E2 reversed these anti-inflammatory effects, and E2 administration to GPER1- knockdown rats had reduced anti-inflammatory effects. As little is known about the effects of estrogens on microglia, even less is known about the effects of progesterone.

P acts on neurons and glia through classical genomic mechanisms by binding to nuclear progesterone receptor (PR), of which there are two isoforms (PRA, PRB), and via non-classical, a.k.a. non-genomic, membrane-bound progesterone receptors [mPRs; (201)]. Tameh et al. (202) found that a mechanism responsible for P's ability to mediate neuronal survival signaling cascades is through upregulation of certain NMDA subunits, specifically GluN1, GluN2A, and GluN3A, which mediate neuronal survival signaling cascades (203, 204). By upregulating these subunits, P serves to inhibit NMDA-mediated apoptosis.

Work by Bali et al. (205) revealed that P exerts its effects on microglia by binding to progesterone receptor membrane component 1 (PGRMC1, a.k.a. 25-Dx, a.k.a. ventral midline antigen or VEMA). PGRMC1 belongs to neither the classical, nor the membrane-bound progesterone receptor subfamilies, but rather the membrane-associated progesterone receptor (MAPR) family (206). PGRMC1 is the P-binding protein in a single-transmembrane protein complex (207–209) and has been found on the membranes of the Golgi apparatus, endoplasmic reticulum, and mitochondria of CNS cells (210, 211). Both E2 and P upregulate PGRMC1 expression in the ovariectomized rat HPC (212). Activating PGRMC1 with P reinstates neuronal activity (213), enhances spinogenesis (214), and enhances neuronal migration and myelination from Schwann cells in the spinal column, where PGRMC1 has been found in the cell membrane (208, 215). What's more, P has been found to induce BDNF expression, an effect which has been shown to be mediated by both the classical PR in cortical slice explants (216), and by PGRMC1 in cultured glial cells (217). This suggests that ketamine, E2, and P, each on their own, would increase BDNF expression. However, this is contradictory to seminal research which showed that P can antagonize E2's synaptogenesis-inducing effects at certain time points (218).

Prior to the discovery that PGRMC1 is crucial for microglial activation (205), work by the same authors showed that P antagonizes E2-mediated neurite outgrowth *in vitro*, but only when microglial cells were also present. P had no effect on neurite outgrowth with only neurons and astrocyte cocultures (219). This line of research culminated in several additional important findings. Firstly, activating PGRMC1 induces microglial activation to the same degree as stimulation from LPS, as indicated by CD11b protein expression. Second, when microglia are activated via P-binding to PGRMC1 they inhibit new neurite outgrowth. Third, that P binding to PGRMC1 inhibits BDNF release from astrocytes, further hindering neuritogenesis. Fourth, PGRMC1 knockdown stopped LPS and injury (*in vitro* scratch-wounding) induced microglia activation (205, 220). Together, these findings suggest that PGRMC1 is critical for microglial activation, and that E2 and P may be acting to antagonize one another, with E2 causing quiescence in microglia, but P activating them. This is of course counter-intuitive when considering P's well-researched neuroprotective effects in the context of ischemia (221–229). Looking to P's broader effects on inflammation could, perhaps, clarify this dichotomy. P also has multiple anti-inflammatory effects in LPS-stimulated microglia *in vitro*, as indicated by Lei et al. (230). LPS upregulated the pro-inflammatory cytokine TNF-α, inducible nitric oxide synthase (iNOS), an enzyme precursor for the free radical nitric oxide (NO), and cyclooxygenase-2 (COX-2), an enzyme precursor for prostaglandin. All of which are upregulated during inflammation. P attenuated these LPS-induced increases in a dose- dependent manner. Additionally, P decreased the LPS-induced phosphorylation of several other kinases such as p38, c-Jun N-terminal kinase, and extracellular regulated kinase MAPKs.

**Figure 2:**
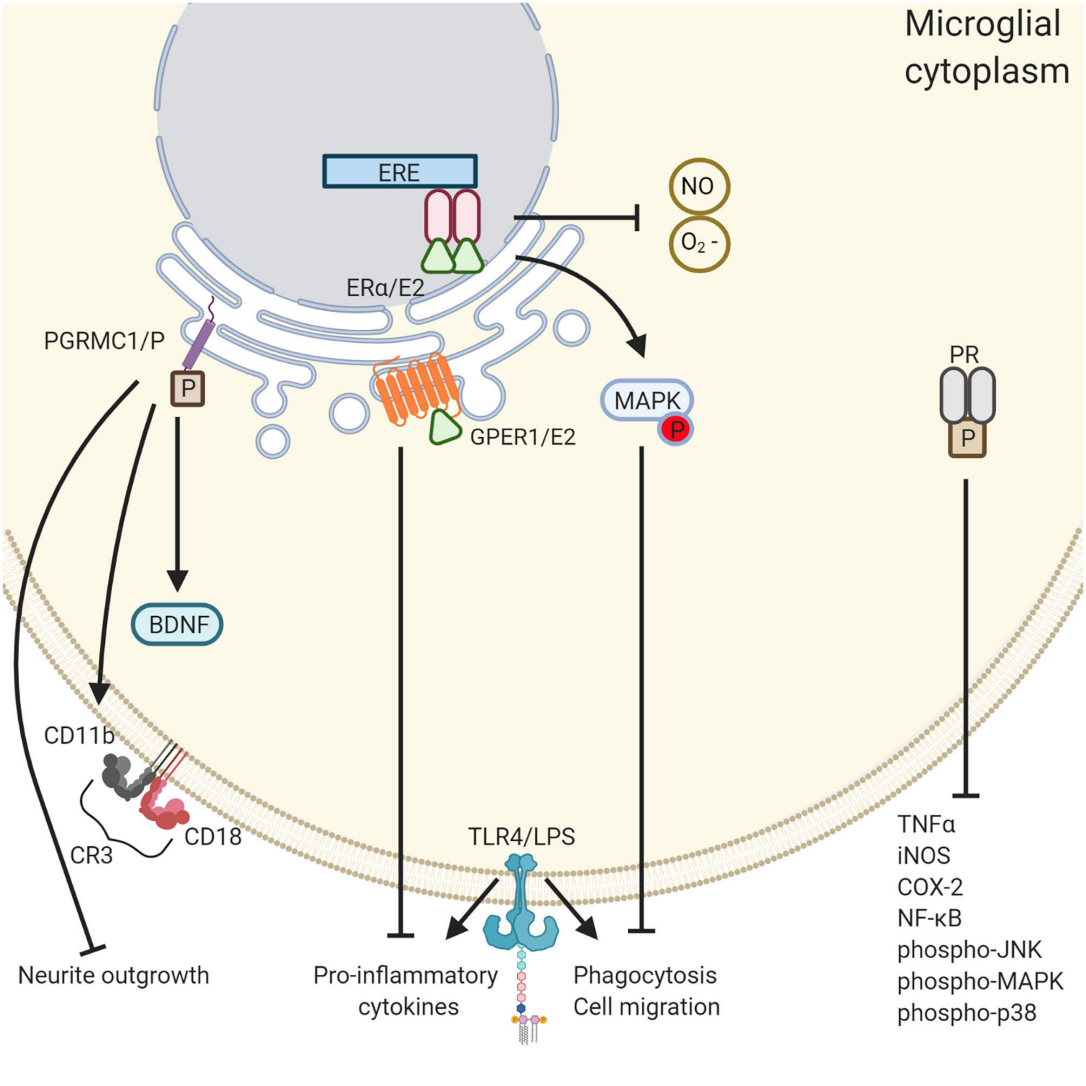
The differential roles of ovarian hormones in the microglial immune response. PGRMC1 activation induces microglial activation whereas PR and E2 quiesces it. PGRMC1 binds to progesterone leading to the inhibition of neurite outgrowth, microglia activation through the expression of CD11b and CD18, and the inhibition of BDNF. Conversely, GPER1 activation by E2 reduced the expression of proinflammatory cytokines TNF-α and IL-1β. In addition, E2 reduces microglial phagocytosis and cell migration through activation of the MAPK pathway and inhibits the release of reactive oxygen species NO and O2^−^. PR activation also has an anti- inflammatory effect through the inhibition of a various of inflammatory cytokines. Created with BioRender.com.

Importantly, P decreased the LPS-induced activation of the protein complex nuclear factor kappa-light-chain-enhancer of activated B cells (NF-κB). NF-κB is a transcription factor which controls the expression of various target genes, especially those involved in immune and inflammatory responses (230). NF-κB and pro-inflammatory cytokines have a bidirectional connection: NF-κB is activated by pro-inflammatory cytokines, and directly promotes pro-inflammatory cytokine production by binding to cytokine promoter regions in the genome (231). These pleiotropic effects of P, along with the PGRMC1 activational mechanism, all specific to microglia, demonstrate the relevance of understanding how P, E2, and ketamine might interact with one another. Of course, much more comprehensive research still needs to be done to understand the complexity of these phenomena–see Figure 2.

## Ketamine Binds to ERα

It has been shown that estrogens act to mediate enhanced sensitivity to ketamine in depression models using rodents (156–158). Furthermore, both female mice (152) and humans (160) metabolize ketamine to produce higher levels of the crucially important ketamine metabolites 2R-6R/2S-6S HNK than males. It is thought that this occurs due to women having higher levels of the CYP2A6 and CYP2B6 enzymes, responsible for ketamine's metabolism (232). E2 and P are capable of inducing these enzymes (233), with E2 doing so via ERα (234). ERα is therefore critical in understanding how ketamine and ovarian hormones might interact. In most tissues, including the CNS, E2 binding to ERs precipitates an upregulation of PRs (235). Thanks to work by Alves et al. (236) it is known that this occurs via E2 binding to ERα receptors in the HPC of wild- type mice (237). Both E2 and P lead to the induction of PGRMC1 in female ovariectomized rat hippocampal neurons in the CA1, CA3, and DG (212), although it is unclear which ER/PR-binding mechanisms cause this.

Evidence that ketamine binds to ERα, was provided by Ho et al. (232). Radioligand binding assays, coupled with surface plasmon resonance, demonstrated that E2, ketamine, and 2R-6R/2S-6S HNK bind to ERα receptors in cultured astrocytes. They also found that the same compounds act in an additive manner to induce AMPA receptor subunits, again *in vitro*. This is thought to be crucial for ketamine's antidepressant effects, as previously discussed, and this effect was ablated by ERα knockdown. Given that all three compounds also lead to ERα trafficking to the nucleus, it follows that the authors hypothesized a potential positive feedback loop. Namely, when ERα binds to estrogen-response elements, CYP2A6, CYP2B6, and AMPA subunit transcription is induced, leading to more ketamine metabolism and more AMPA receptors (232). Research has yet to be conducted to determine how PRs might be impacted by these processes but it is likely that, via ERα binding, ketamine and its metabolites cause PR upregulation. Virtually nothing is known of ketamine's ability to impact PGRMC1. How this might be manifested behaviorally is an even greater mystery.

## Hippocampus As a Candidate for Ketamine-Hormone Interactions

Depression involves multiple brain regions. The HPC is perhaps the most well-studied brain region in regards to MDD. A meta-analysis of MRI results yielded an average reduction of 8–10% in HPC volume among individuals with unipolar depression (238). The HPC is also a site of continuous neurogenesis as documented in adult macaques (239). That is, while depression may decrease HPC volume, this change may not necessarily be permanent. Outside the context of antidepressant research, it is worth noting that HPC volume declines naturally with age, and that there exists an interactive effect of gender and age on HPC volume decline. As we age, HPC volume declines linearly among men, whereas there is no such correlation among women (240).

Morphological changes in the HPC such as neurogenesis and synaptogenesis are, in part, mediated by BDNF (59, 241, 242). What's more, researchers have shown that peripheral BDNF leads to both hippocampal neurogenesis among adult mice, as well as antidepressant-like effects in the forced swim test [FST; (75)]. Ketamine is well-documented to both increase hippocampal BDNF, and produce antidepressant-like effects in the FST in male rodents (243–246). Furthermore, it is likely that the neuroprotective effects of E2 are partly due to BDNF upregulation (247). Some speculate that the BDNF gene contains an estrogen response element such that E2 could upregulate BDNF directly (248). Others have shown that estrogen receptors are found on BDNF-expressing neurons (249, 250). It is perhaps through this mechanism that E2 mediates dendritic spine growth and synaptogenesis in the HPC, as previously discussed (218, 251).

**Figure 3:**
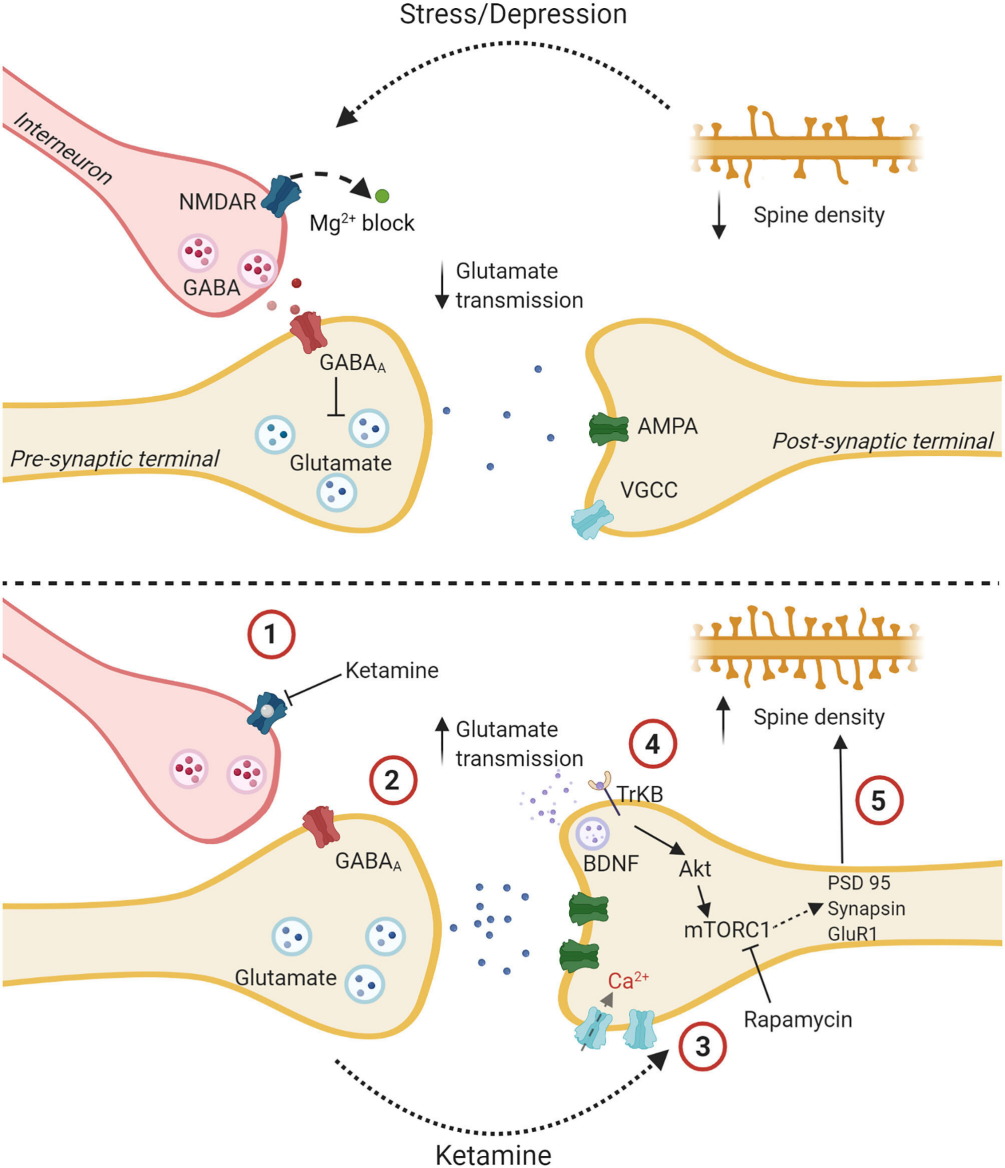
The glutamate burst hypothesis: a proposed mechanism of action for ketamine. The upper panel shows a glutamatergic synapse tonically inhibited by a GABAergic interneuron with decreased spine density and altered glutamatergic transmission due to stress and depression. The lower panel delineates putative steps for ketamine's antidepressant effect. (1) Ketamine binds to an NMDA receptor on a GABAergic interneuron. (2) This reduces the tonic inhibition of a glutamatergic presynaptic terminal which causes the release of bursts of glutamate. (3) Increased signal transmission leads to upregulation of AMPA receptors, increased depolarization, and influx of Ca^2+^ through Voltage gated calcium channels. (4) Higher intracellular Ca^2+^ leads to the release of BDNF which binds to the TrKB receptor. The Akt pathway is activated followed by the mTOR pathway. (5) This leads to the synthesis of synaptic proteins and increased spine density. Created with BioRender.com.

What makes the HPC particularly interesting in terms of ketamine's effects is, as previously discussed, that the HPC-PFC circuits are likely the most crucial for ketamine's antidepressant effect. Within this circuit ketamine selectively disinhibits GABA interneurons, as per the glutamate burst hypothesis—see Figure 3. With regards to the neuroinflammatory hypothesis, ovarian hormones are known to play an important role in the inflammatory response in the HPC (252, 253) and prefrontal cortex (254). The HPC has been the focus of several research groups who found that ketamine generally suppresses glial inflammation in this area (177, 255, 256).

## Conclusion

The etiology of major depressive disorder is complex, and there is no single explanation for its manifestation. Likewise, it's possible that there are many valid explanations as to why women suffer from MDD more than men, but ovarian hormones are surely implicated. Despite the discovery of ketamine's antidepressant effects being relatively recent, there's plenty of evidence supporting the various hypotheses concerning its mechanisms of action in the CNS. However, evidence specific to the female sex is lacking. That which has been gathered only demonstrates that well-researched theories do not generalize across the sexes. Considering the neuroinflammatory hypothesis of depression may help shed light on ketamine's mechanism of action among females. Indeed, a massive body of literature supports the relevance of this hypothesis, particularly the role of microglia, to novel antidepressants like ketamine. The literature provides more than sufficient evidence to warrant the idea that circulating E2 and P would interact with ketamine's effect on the CNS. In believing some, one might suspect that the three molecules would act additively to reduce depressive symptoms. Believing other lines of research, one might expect E2 to act additively with ketamine, but that P would antagonize this via acting on PGRMC1 on microglia. Evidence which indicates P has neuroinflammatory effects is contradicted by evidence which shows it has anti-inflammatory effects. *In vivo* experimentation looking specifically at the HPC is the next step in investigating these complex phenomena, as much of the literature uses *in vitro* models. Exploring this is paramount to the likely eventuality that ketamine, or a similar compound, will be administered to those seeking treatment for MDD on a larger scale.

## Author Contributions

CG wrote the original manuscript as part of his Masters thesis. AP contributed to sections of the manuscript and edited later versions (including figures). WB supervised the process and edited the final document. All authors contributed to the article and approved the submitted version.

## Funding

This work was supported by the Natural Sciences and Engineering Research Council of Canada (NSERC).

## Conflict of Interest

The authors declare that the research was conducted in the absence of any commercial or financial relationships that could be construed as a potential conflict of interest.

## Publisher's Note

All claims expressed in this article are solely those of the authors and do not necessarily represent those of their affiliated organizations, or those of the publisher, the editors and the reviewers. Any product that may be evaluated in this article, or claim that may be made by its manufacturer, is not guaranteed or endorsed by the publisher.
